# Regulation of neuronal excitation–transcription coupling by Kv2.1-induced clustering of somatic L-type Ca^2+^ channels at ER-PM junctions

**DOI:** 10.1073/pnas.2110094118

**Published:** 2021-11-08

**Authors:** Nicholas C. Vierra, Samantha C. O’Dwyer, Collin Matsumoto, L. Fernando Santana, James S. Trimmer

**Affiliations:** ^a^Department of Physiology and Membrane Biology, University of California Davis School of Medicine, Davis, CA 95616

**Keywords:** calcium signaling, membrane contact sites, excitation–transcription coupling, voltage-gated calcium channels, voltage-gated potassium channels

## Abstract

In mammalian brain neurons, membrane depolarization leads to voltage-gated Ca^2+^ channel-mediated Ca^2+^ influx that triggers diverse cellular responses, including gene expression, in a process termed excitation–transcription coupling. Neuronal L-type Ca^2+^ channels, which have prominent populations on the soma and distal dendrites of hippocampal neurons, play a privileged role in excitation–transcription coupling. The voltage-gated K^+^ channel Kv2.1 organizes signaling complexes containing the L-type Ca^2+^ channel Cav1.2 at somatic endoplasmic reticulum–plasma membrane junctions. This leads to enhanced clustering of Cav1.2 channels, increasing their activity. However, the downstream consequences of the Kv2.1-mediated regulation of Cav1.2 localization and function on excitation–transcription coupling are not known. Here, we have identified a region between residues 478 to 486 of Kv2.1’s C terminus that mediates the Kv2.1-dependent clustering of Cav1.2. By disrupting this Ca^2+^ channel association domain with either mutations or with a cell-penetrating interfering peptide, we blocked the Kv2.1-mediated clustering of Cav1.2 at endoplasmic reticulum–plasma membrane junctions and the subsequent enhancement of its channel activity and somatic Ca^2+^ signals without affecting the clustering of Kv2.1. These interventions abolished the depolarization-induced and L-type Ca^2+^ channel-dependent phosphorylation of the transcription factor CREB and the subsequent expression of c-Fos in hippocampal neurons. Our findings support a model whereby the Kv2.1-Ca^2+^ channel association domain-mediated clustering of Cav1.2 channels imparts a mechanism to control somatic Ca^2+^ signals that couple neuronal excitation to gene expression.

## Significance

In hippocampal neurons, gene expression is triggered by electrical activity and Ca^2+^ entry via L-type Cav1.2 channels in a process called excitation–transcription coupling. We identified a domain on the voltage-gated K^+^ channel Kv2.1 that promotes the clustering of L-type Cav1.2 channels at endoplasmic reticulum–plasma membrane junctions in the soma of neurons. Importantly, we discovered by disrupting this domain that the Kv2.1-mediated clustering of Cav1.2 at this somatic microdomain is critical for depolarization-induced excitation–transcription coupling.

In brain neurons, Ca^2+^ influx through L-type voltage-gated Ca^2+^ channels (LTCCs) initiates diverse physiological responses, including the regulation of membrane potential, release of intercellular signaling molecules, and changes in gene expression (1–3). Cav1.2 is the major LTCC principal α_1_ subunit expressed in hippocampal neurons (4–8), and Cav1.2 knockout mice have deficits in hippocampal long-term potentiation, memory, and related behaviors (6, 9–11). Neurons organize Cav1.2-containing LTCCs into microdomains containing different effectors that enable Ca^2+^ influx through these channels to activate specific Ca^2+^ signaling pathways (12–14). Extensive studies of Cav1.2 channels on distal dendrites and dendritic spines have defined mechanisms whereby Cav1.2-mediated Ca^2+^ influx leads to short- and long-term synaptic plasticity and activity-dependent gene expression, including their coupling to signaling proteins key to transcription factor activation (14–21). However, relatively little is known of the mechanisms controlling the subcellular localization and function of the prominent population of Cav1.2 channels found in the plasma membrane (PM) of the neuronal cell body (or soma), the “aspiny” regions of hippocampal neurons (22), or their role in responses mediated by LTCC-dependent Ca^2+^ signaling.

The voltage-gated K^+^ channel Kv2.1 is present in high-density clusters on the soma and proximal dendrites of most brain neurons (23–28) and is not detectable in distal dendrites or in dendritic spines (24, 25). Kv2.1 is clustered at PM sites that form contacts with endoplasmic reticulum (ER), termed ER-PM junctions, that are formed and stabilized by the association of PM Kv2.1 with the ER membrane–resident proteins VAPA and VAPB (29, 30). This structural function of Kv2.1 is independent of its K^+^-conducting function (31) and requires the phosphorylation-dependent binding of Kv2.1 to VAP proteins via a PRC domain located in Kv2.1’s extensive C terminus (29, 30). Mutations in Kv2.1 predicted to disrupt the PRC domain cause severe neurological disease in humans (32, 33), suggesting an important physiological role for Kv2.1 channel–induced ER-PM junctions in normal brain function. In general, ER-PM junctions function as specialized organizing domains for protein complexes mediating Ca^2+^ and lipid signaling and homeostasis (34).

We recently identified Cav1.2 as a protein associated with Kv2.1-containing ER-PM junctions (35). We found that in hippocampal pyramidal neurons, Kv2.1 promotes the increased clustering and activity of Cav1.2 channels and their localization at somatic ER-PM junctions (35). At these sites, PM clusters of Kv2.1 and Cav1.2 juxtapose with ER-localized ryanodine receptor (RyR) Ca^2+^ release channels (26, 36). Ca^2+^ influx through Cav1.2 triggers the opening of RyRs, producing spontaneous localized Ca^2+^ elevations (Ca^2+^ sparks) which amplify the Ca^2+^ signal beyond that obtained with Cav1.2 activation alone (35). In addition to promoting the coupling of Cav1.2 with RyRs and other signaling proteins, the Kv2-mediated clustering of Cav1.2 channels also increases Cav1.2 open probability (35, 37). However, neither the molecular determinants of LTCC targeting to somatic Kv2-associated ER-PM junctions nor the physiological consequences of Ca^2+^ signals produced at these sites are known.

Here, we identified a Ca^2+^ channel association domain (CCAD) within Kv2.1’s cytoplasmic C terminus that is necessary for Cav1.2 localization to Kv2.1-containing ER-PM junctions. The disruption of the Kv2.1 CCAD selectively impairs the ability of Kv2.1 to recruit Cav1.2 to ER-PM junctions but does not impact Kv2.1 clustering at these sites. A cell-penetrating interfering peptide based on the CCAD sequence caused the spatial and functional decoupling of Cav1.2 from Kv2.1-associated ER-PM junctions and significantly reduced Cav1.2 channel activity and LTCC-dependent gene expression in neurons. Our data provide additional evidence that Cav1.2 channels on neuronal somata play a prominent role in excitation–transcription coupling and that the Kv2.1-dependent regulation of the clustering, activity, and localization of this population of Cav1.2 channels shapes their physiological function.

## Results

### Identification of a Ca^2+^ Channel Association Domain in the Cytoplasmic C Terminus of Kv2.1.

**Fig. 1.**
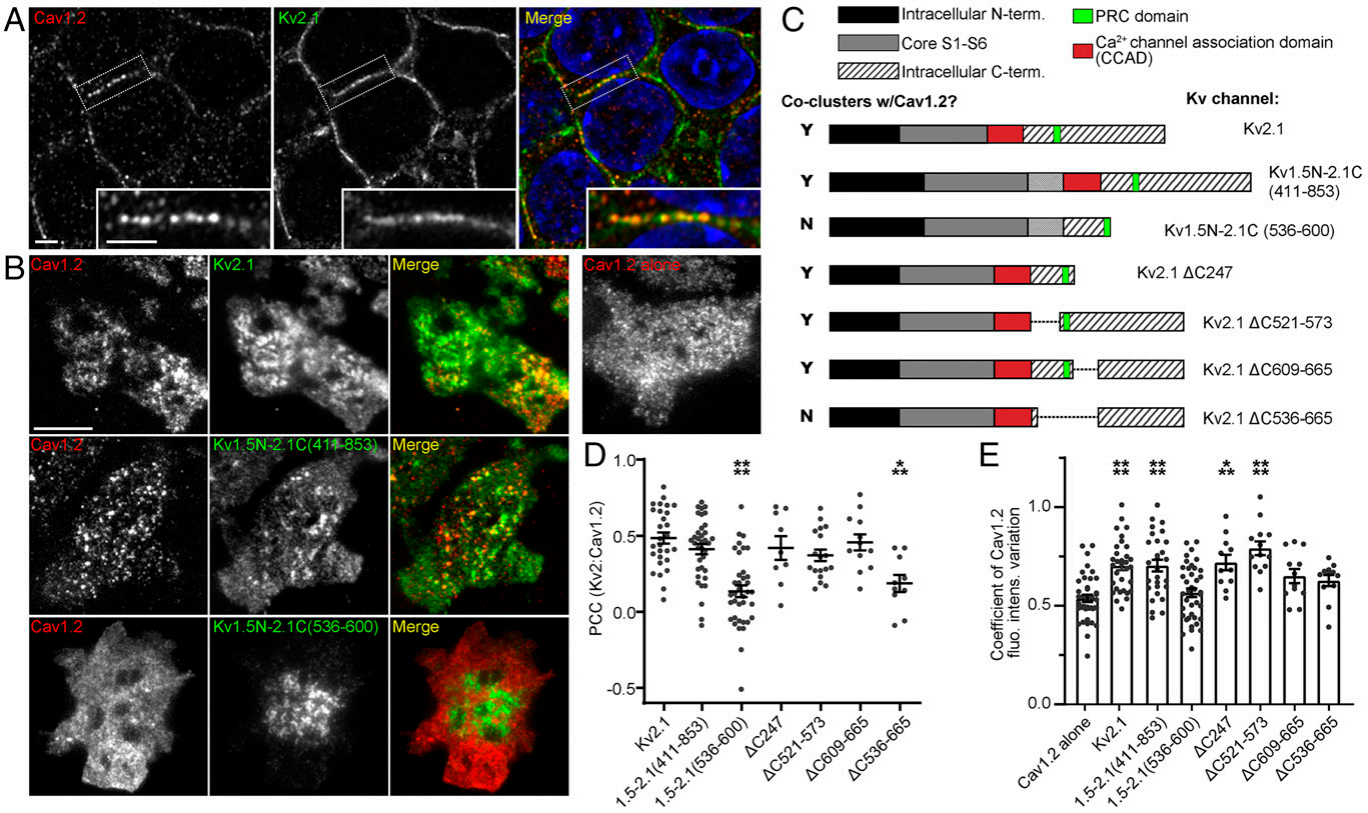
Identification of a domain within the C terminus of Kv2.1 required for coclustering with Cav1.2. (*A*) Confocal optical section of the dentate gyrus granule cell layer acquired from an adult mouse brain section immunolabeled for Cav1.2 (red) and Kv2.1 (green). Hoechst staining (blue) is shown in the merge panel (Scale bar, 2 μm in both the main panel and the magnified *Inset* whose location in the main panels are outlined.) (*B*) TIRF images of HEK293T cells transfected with Cav1.2 (red) and WT and chimeric Kv2.1 constructs (green) (Scale bar, 10 μm and holds across all panels). (*C*) Diagram of Kv2.1 chimeras, and truncation, and internal deletion mutants used to identify the region in Kv2.1 required for the reorganization of Cav1.2 channels. In the left column, coclustering of WT and chimeric Kv2.1 constructs with Cav1.2 is indicated by Y (yes) or N (no), as determined by displaying a significantly lower PCC value as compared to Kv2.1 WT. Within the region encoding the Kv2.1 C terminus, the putative CCAD is highlighted in red, and the PRC domain in green. (*D*) PCC values of the fluorescence signals of WT and chimeric Kv2 constructs and Cav1.2 channel fluorescence signals expressed in HEK293T cells. Each point represents a single cell; bars are mean ± SEM (****P < 0.0001 and ***P = 0.0009; one-way ANOVA followed by Dunnett’s post hoc test versus Kv2.1 WT). (*E*) CV values of Cav1.2 channel fluorescence when coexpressed with Kv2 channels in HEK293T cells. Each point represents a single cell; bars are mean ± SEM (****P < 0.0001 and ***P < 0.0006; one-way ANOVA followed by Dunnett’s post hoc test versus Cav1.2 alone).

Cav1.2 channels colocalize with Kv2.1 channels in the somata of hippocampal neurons (35), as illustrated by confocal images obtained from a mouse brain section immunolabeled with antibodies against Kv2.1 and Cav1.2 (Fig. 1*A*). When Cav1.2 channels are expressed in human embryonic kidney (HEK) 293T cells, they also cluster with Kv2.1 at ER-PM junctions (Fig. 1*B*). To define the molecular requirements of Kv2.1-mediated rearrangement of Cav1.2 channels, we coexpressed Cav1.2 tagged with monomeric RFP [Cav1.2-RFP (38)], with a series of previously described Kv2.1 deletion mutants and chimeras (39, 40) with altered Kv2.1 C-terminal domains (Fig. 1*C*) in HEK293T cells. We used total internal reflection fluorescence (TIRF) microscopy to image the PM and near-PM populations of the immunolabeled and fluorescent proteins. We determined the degree of colocalization between the Kv2.1 isoforms and Cav1.2 by measuring Pearson’s correlation coefficient (PCC) values of the respective fluorescence signals (Fig. 1*D*). As a quantitative index of Cav1.2 channel clustering, we used the coefficient of variation of Cav1.2-RFP fluorescence intensity (CV, obtained by dividing the SD of the fluorescence signal by the mean fluorescence signal). A larger CV value indicates a higher degree of channel clustering (27).

Similar to previous results (35), we found that full-length Kv2.1 colocalized with Cav1.2-RFP channels, as indicated by high PCC values (Fig. 1*D*). Coexpression with Kv2.1 also resulted in the enhanced clustering of Cav1.2 channels, as indicated by the increased Cav1.2 CV values upon Kv2.1 coexpression relative to control values (Fig. 1*E*). We next investigated whether the domain underlying the Kv2.1-mediated recruitment of Cav1.2 to ER-PM junctions was located on the cytoplasmic C terminus of Kv2.1 by evaluating a chimeric protein containing the entire Kv2.1 C terminus (amino acid residues 411 to 853 of rat Kv2.1) appended onto the unrelated Kv1.5 channel (Kv1.5N-2.1C [411 to 853]). Kv1.5 itself neither localizes to ER-PM junctions (29) nor impacts the distribution of Cav1.2 channels (35). Similar to full-length Kv2.1 wild-type (WT), the Kv1.5N-Kv2.1C (411 to 853) chimera, which localizes to ER-PM junctions (29), coclustered with Cav1.2 channels and increased Cav1.2 CV values relative to Cav1.2 alone (Fig. 1 *B*, *D*, and *E*). This result demonstrated that the cytoplasmic C terminus of Kv2.1 contained determinants sufficient to both mediate its clustering at ER-PM junctions and to stabilize clustered Cav1.2 channels at these sites.

To further refine which region(s) within the Kv2.1 C terminus underlies the reorganization of Cav1.2 channels, we next used a chimeric protein in which a much smaller portion of the Kv2.1 C terminus (amino acid residues 536 to 600, which contain the PRC domain) was appended to Kv1.5. Although the Kv1.5N-2.1C (536 to 600) chimera still exhibits Kv2.1-like clustering at ER-PM junctions (29) due to the presence of an intact PRC domain (Fig. 1 *B* and *C*), it was incapable of recruiting Cav1.2 channels. The evaluation of a set of Kv2.1 C-terminal internal deletion and truncation mutants supported that the PRC-dependent formation of Kv2.1-organized ER-PM junctions was necessary but not sufficient for the Kv2.1-mediated reorganization of Cav1.2 channels and defined a CCAD between residues 411 and 520 of the Kv2.1 C terminus required for Kv2.1 to reorganize Cav1.2 channels (Fig. 1*C*).

**Fig. 2.**
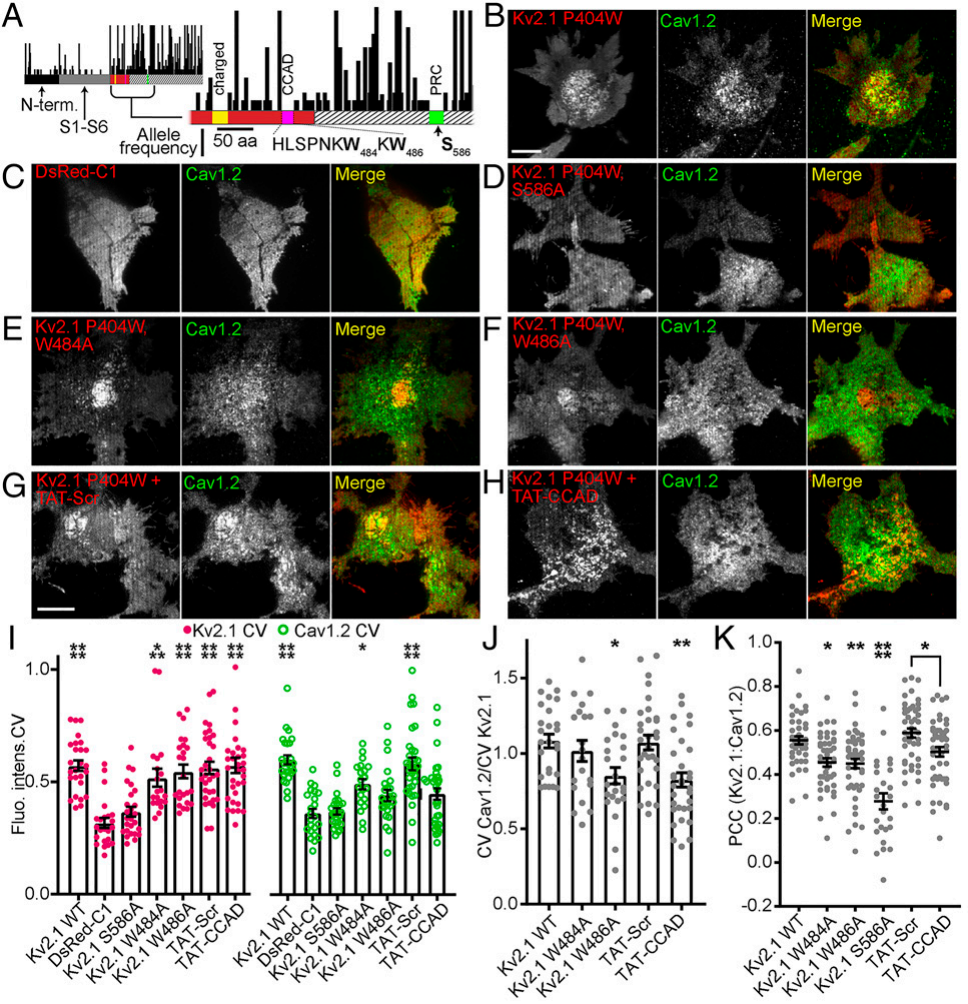
Point mutants and cell-permeant fragments of the Kv2.1 CCAD disrupt the coclustering of Cav1.2 with Kv2.1 in HEK293T cells. (*A*) Plot of nonsynonymous genic variation in the human *KCNB1* gene derived from genome and whole-exome sequences from over 141,000 individuals in the Broad Institute Exome Aggregation Consortium database. Within the expanded view of the region encoding the Kv2.1 C terminus, a charged domain involved in Kv2.1 surface expression is highlighted in yellow, the putative CCAD in red, the genic-intolerant region within the CCAD in pink, and the PRC domain in green. (*B*–*H*) TIRF images of HEK293T cells expressing WT and CCAD point mutant DsRed-Kv2.1 constructs (red) and Cav1.2-GFP (green) (Scale bar, 10 μm and holds for all images). (*I*) CV of Kv2.1 WT and CCAD point mutant (magenta) and Cav1.2 (green) fluorescence values. Each point represents a single cell; bars are mean ± SEM (****P < 0.0001, ***P = 0.0004, and *P = 0.0135 versus negative control DsRed-C1; one-way ANOVA followed by Tukey’s post hoc test). (*J*) Ratio of Cav1.2 CV values to Kv2.1 CV values. Each point represents a single cell; bars are mean ± SEM (**P = 0.0017 and *P = 0.0119; one-way ANOVA followed by Dunnett’s post hoc test versus positive control Kv2.1 WT). (*K*) PCC values of Kv2.1 constructs and Cav1.2-GFP fluorescence signals. Each point represents a single cell; bars are mean ± SEM (****P < 0.0001, **P = 0.0091, and *P = 0.0119 versus positive control Kv2.1 WT; *P = 0.0469 versus TAT-Scr; one-way ANOVA followed by Tukey’s post hoc test).

To gain insights into this putative CCAD-containing region of Kv2.1, we determined the frequency of nonsynonymous polymorphisms in the coding region of the human *KCNB1* (Kv2.1) gene in genomic and exome sequences from over 141,000 individuals (https://gnomad.broadinstitute.org/). The lack of genic variation within a region is indicative of functional importance (41). There is little variation in regions that encode domains critical to the function of Kv2.1 as a voltage-sensing and K^+^-conducting channel (Fig. 2*A*). Conversely, the C terminus of Kv2.1 displays much more genic variation, with the exception of the invariant PRC domain (residues 584 to 597) and a charged domain within the proximal C terminus region important for the cell surface expression of Kv2.1 [within residues 444 to 477 (42)]. A closer inspection of the region-encoding residues 477 to 520 of the Kv2.1 C terminus revealed an additional invariant domain corresponding to residues 475 to 493 of human Kv2.1 (Fig. 2*A*). This same region of Kv2.1 has previously been identified to mediate interaction with the vesicle fusion SNARE protein syntaxin-1A (Stx-1A) in vitro and in neuroendocrine and pancreatic beta cells (43–45). However, whether this region of Kv2.1 is also involved in regulating the subcellular distribution and function of Cav1.2 had not been experimentally addressed.

### Point Mutants and Interfering Fragments of the Kv2.1 CCAD Disrupt Cav1.2 Channel Clustering with Kv2.1.

A previous investigation of the Stx-1A binding domain of Kv2.1 revealed a critical role for tryptophan residues at positions 484 and 486 in mediating the ability of a Kv2.1-derived synthetic peptide to bind Stx-1A in vitro (46). Given the high-sequence conservation of W484 and W486 across vertebrates, the lack of genic variation at these sites in Kv2.1 in the human population, and the results of our Kv2.1 chimera and deletion mutant experiments, we tested whether mutating these same residues would also impact the ability of Kv2.1 to regulate the subcellular organization of Cav1.2. We introduced either the W484A or W486A point mutations in a DsRed-tagged, non–K^+^-conducting point mutant of Kv2.1 (DsRed-Kv2.1 P404W). We chose to use this nonconducting mutant background as the ability of Kv2.1 to organize ER-PM junctions (31) and to increase Cav1.2 localization at these sites is independent of its ability to conduct K^+^ (35), and using this mutant avoids any additional and indirect contributions of the K^+^-conducting function of Kv2.1, for example on membrane potential, to its regulation of Cav1.2. We will hereafter refer to this nonconducting point mutant as “Kv2.1 WT,” in reference to its WT cytoplasmic C terminus.

When expressed in HEK293T cells alone, Kv2.1 WT and the W484A and W486A point mutants showed similar overall expression and PM clustering (*SI Appendix*, Fig. S1). We used TIRF imaging to evaluate the ability of these mutants to colocalize with and cluster coexpressed Cav1.2-GFP (Fig. 2 *B*–*H*). Similar to our previous observations (35), Kv2.1 WT colocalized with Cav1.2-GFP and increased its PM clustering (Fig. 2*B*) relative to Cav1.2-GFP expressed with DsRed alone (Fig. 2*C*). Neither increased colocalization nor clustering of Cav1.2 was seen upon coexpression with a Kv2.1 PRC domain point mutant (S586A) (Fig. 2*D*) that itself does not cluster at ER-PM junctions (29). Kv2.1 constructs containing the W484A or W486A mutations still clustered like Kv2.1 WT (Fig. 2 *E* and *F*), which was expected as they retain an intact PRC domain. However, when compared to Kv2.1 WT, these mutants were deficient in their ability to recruit and reorganize Cav1.2-GFP channels, as indicated by the reduced Cav1.2 PCC and CV values relative to Kv2.1 WT (Fig. 2 *I*–*K*). Together, these results demonstrate that W484 and W486 are necessary for the Kv2.1-dependent reorganization of Cav1.2 channels.

We next tested how the introduction of a cell-penetrating peptide, containing amino acids (a.a.) 478 to 486 of the putative Kv2.1 CCAD, impacted the ability of Kv2.1 to recruit Cav1.2 channels in HEK293T cells. This peptide [TAT-CCAD, also called TAT-C1aB (44)] contains these 9 a.a. of Kv2.1 fused to a TAT motif that directs its efficient uptake into cells following its addition to culture medium (47). The overnight treatment of HEK293T cells coexpressing Kv2.1 WT and Cav1.2-GFP with TAT-CCAD reduced coclustering of Kv2.1 and Cav1.2 without disrupting Kv2.1 clustering itself (Fig. 2 *G*–*K*). A version of this peptide in which the Kv2.1 sequence was scrambled (TAT-Scr) had no impact on the ability of Kv2.1 WT to reorganize Cav1.2 (Fig. 2 *G*–*K*). These results support that the CCAD found within a.a. 478 to 486 in Kv2.1 is both necessary and sufficient for the ability of Kv2.1 to increase the clustering of Cav1.2.

### The Kv2.1 CCAD Is Required for Kv2.1-Mediated Increases in Cav1.2 Clustering and Function.

**Fig. 3.**
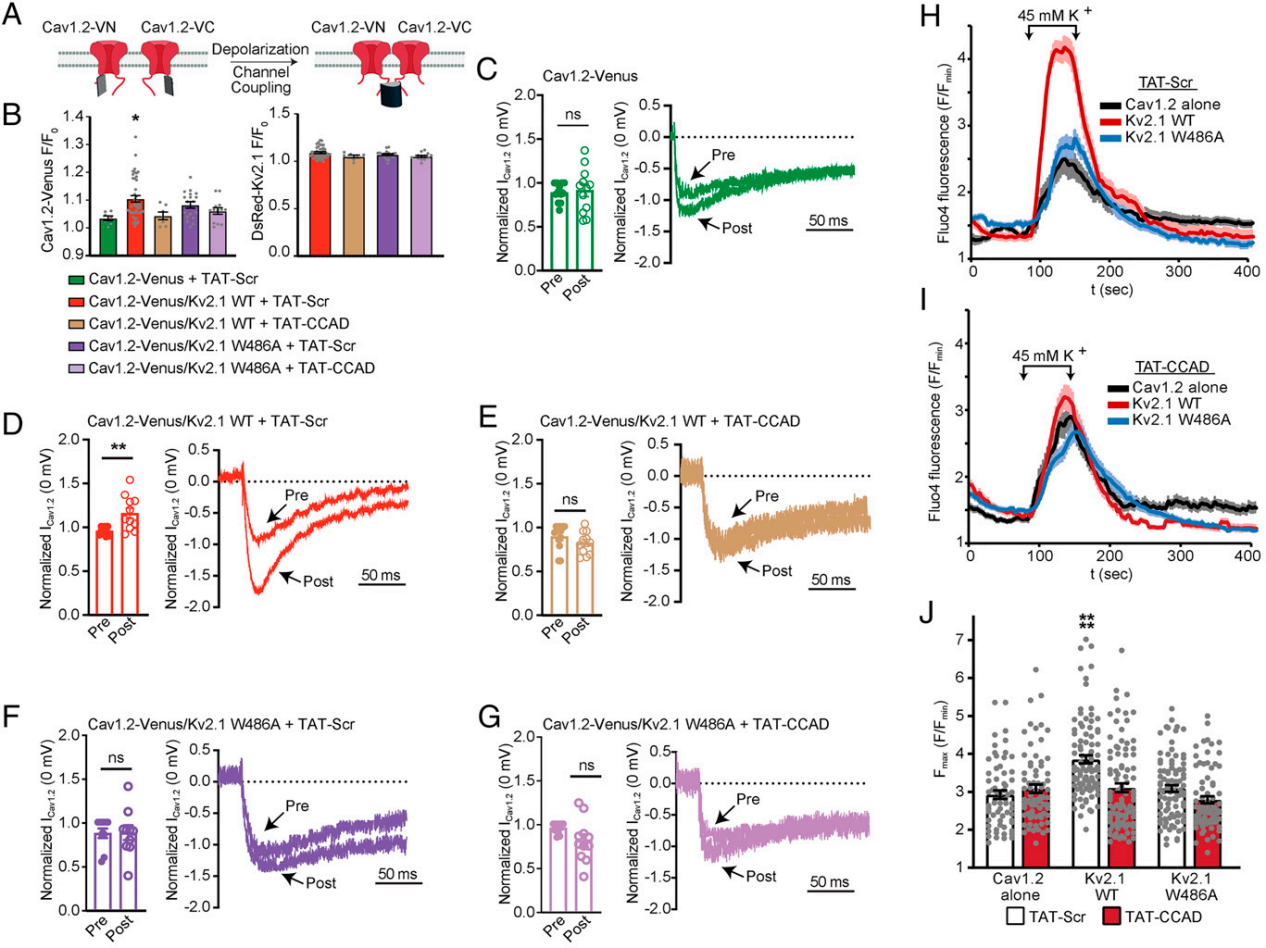
Point mutants and cell-permeant fragments of the Kv2.1 CCAD impair Kv2.1-dependent enhancement of Cav1.2 coclustering and LTCC function. (*A*) Illustration of Cav1.2 fused to the VN and VC fragments of the split-Venus bimolecular complementation system and its use to report on the impact of depolarization on Cav1.2 channel coupling. (*B*) Cav1.2-Venus and DsRed-Kv2.1 fluorescence (F/F_0_) acquired postdepolarization from TIRF images of transfected HEK293T cells treated with TAT-Scr or TAT-CCAD (1 μM) peptides. Each point represents a single cell; bars are mean ± SEM (*P = 0.0486 versus Cav1.2-Venus + TAT-Scr; one-way ANOVA followed by Tukey’s post hoc test). (*C–G*) Mean *I*_Ca_ and representative currents measured at 0 mV pre- and postconditioning from HEK293T cells expressing the indicated constructs and treated with TAT-Scr or TAT-CCAD. Each point represents a single cell; bars are mean ± SEM (**P = 0.0094 and ns: not significant; paired *t* test). (*H* and *I*) Representative Fluo-4 fluorescence intensity measurements obtained from HEK293T cells transfected with indicated constructs and treated with TAT-Scr or TAT-CCAD peptides. Ca^2+^ influx was stimulated by depolarization resulting from 60 s exposure to medium containing 45 mM K^+^, as indicated on the graph. Lines are average ± SEM. (*J*) Peak fluorescence values (F/F_min_) obtained during 45 mM K^+^-induced depolarization of Fluo-4–loaded HEK293T cells. Each point represents a single cell; bars are mean ± SEM (****P < 0.0001; one-way ANOVA followed by Dunnett’s post hoc test versus Cav1.2 alone).

We next applied a bimolecular fluorescence complementation approach to test the hypothesis that as the TAT-CCAD peptide disrupts Kv2.1-Cav1.2 association, it would interfere with the previously demonstrated ability of Kv2.1 to increase Cav1.2 coclustering and function (35, 37). For these experiments, we used a system employing Cav1.2 channels fused to the N- or C terminus of the split-Venus fluorescent protein, consisting of Cav1.2-VN155(I152L) and Cav1.2-VC155, respectively. VN155(I152L) and VC155 do not fluoresce in isolation, but they reconstitute the fluorescent Venus protein when brought into close proximity by interaction of the separately tagged Cav1.2 subunits (38). We have used these reporters to show that depolarization-induced Ca^2+^ influx through Cav1.2 channels increases spontaneous Cav1.2-Cav1.2 interactions (38, 48), as shown in Fig. 3*A*, and that coexpression with Kv2.1 further increases the probability of Cav1.2-Cav1.2 channel interactions (37). Here, we found that the application of TAT-CCAD reduced the Kv2.1-dependent increases in Cav1.2-Venus fluorescence intensity without affecting the intrinsic fluorescence of DsRed-Kv2.1 itself (Fig. 3*B*). In contrast, cells treated with the control TAT-Scr peptide exhibited typical Kv2.1-mediated increases in Cav1.2 split-Venus fluorescence (Fig. 3*B*).

These results indicated that the TAT-CCAD peptide selectively disrupted the ability of Kv2.1 to increase Cav1.2-Cav1.2 interactions. Based on our observation that the Kv2.1 W484A and W486A mutants were impaired in their ability to reorganize Cav1.2 channels, we hypothesized that the TAT-CCAD cell-penetrating peptide competed with the CCAD on Kv2.1 for binding to Cav1.2 or to an intermediary protein that promotes Cav1.2 localization at ER-PM junctions. Because the Kv2.1 W484A and W486A mutants would already be deficient for this interaction, we predicted that we would observe no additional effect of the TAT-CCAD peptide on Cav1.2-Cav1.2 interactions in cells expressing Kv2.1 CCAD mutants. For these experiments, we used the Kv2.1 W486A mutant, which displayed a larger reduction in its ability to reorganize Cav1.2 than did Kv2.1 W484A (Fig. 2 *I* and *J*). Cells expressing the Kv2.1 W486A point mutant did not display depolarization-induced elevations in split-Venus fluorescence, as seen for Kv2.1 WT (Fig. 3*B*). Moreover, neither the TAT-CCAD nor the TAT-Scr peptide had any additional effect on Cav1.2 Venus fluorescence in Kv2.1 W486A-expressing cells. These results support the model that an intact Kv2.1 CCAD domain is necessary for the Kv2.1-mediated increase in Cav1.2-Cav1.2 interactions, and that the TAT-CCAD cell-penetrating peptide acts as a competitive inhibitor of the direct or indirect association of Kv2.1 with Cav1.2 via this domain.

While collecting reconstituted fluorescence data in the split-Venus experiments, we also determined how disrupting the Kv2.1 CCAD impacted Cav1.2-Venus channel Ca^2+^ currents. Cav1.2 currents increase in parallel with stimulus-induced increases in Cav1.2-Cav1.2 interactions (38, 48) (Fig. 3*C*) and are further potentiated by Kv2.1-mediated clustering (37) (Fig. 3*D*). We found that the treatment of cells with the TAT-CCAD peptide, but not the TAT-Scr peptide, prevented these stimulus-induced increases in Cav1.2 currents (Fig. 3 *D* and *E*). Cells expressing the Kv2.1 W486A variant also exhibited a smaller increase in Ca^2+^ currents relative to those observed in postdepolarization conditioning in the presence of Kv2.1 WT (Fig. 3 *F* and *G*). Treatment with either of the TAT peptides produced no further effects in the Kv2.1 W486A-expressing cells (Fig. 3 *F* and *G*). Overall, the impact of these manipulations on Cav1.2 Ca^2+^ currents paralleled their effects on Cav1.2-Cav1.2 interactions as reported by split-Venus fluorescence.

Consistent with its role in enhancing Cav1.2 channel activity, coexpression with Kv2.1 also enhances depolarization-induced Ca^2+^ influx in Cav1.2-expressing HEK293T cells, as measured with the Ca^2+^-sensitive dye Fluo-4 (35). To test whether the TAT-CCAD peptide would have any direct effects on Cav1.2 function in the absence of Kv2.1, we compared the effects of TAT-CCAD treatment on depolarization-induced Ca^2+^ influx in Cav1.2-expressing HEK293T cells in the presence and absence of Kv2.1 coexpression. We found that the TAT-CCAD peptide impaired the Kv2.1-dependent increases in depolarization-evoked Ca^2+^ influx in HEK293T cells relative to cells with no treatment or treated with the TAT-Scr peptide (Fig. 3 *H*–*J*). However, neither the TAT-CCAD nor TAT-Scr peptides had any effect on Ca^2+^ influx in cells expressing Cav1.2 alone, showing that these peptides impacted Cav1.2 function in a Kv2.1-dependent manner. As expected from the results of the Cav1.2-Venus experiments, Kv2.1 W486A did not increase Cav1.2-mediated Ca^2+^ influx as did Kv2.1 WT, and TAT-CCAD treatment did not impact Ca^2+^ influx in cells expressing Kv2.1 W486A. These observations show that the impact of TAT-CCAD on Cav1.2 function requires the coexpression of Kv2.1 WT. Taken together, these data further support that Kv2.1 channels promote Cav1.2 clustering and activity and that an intact Kv2.1 CCAD is required for this effect.

### Disruption of the Kv2.1 CCAD Reduces Endogenous Cav1.2 Channel Activity and Its Clustering with Kv2.1 at ER-PM Junctions.

**Fig. 4.**
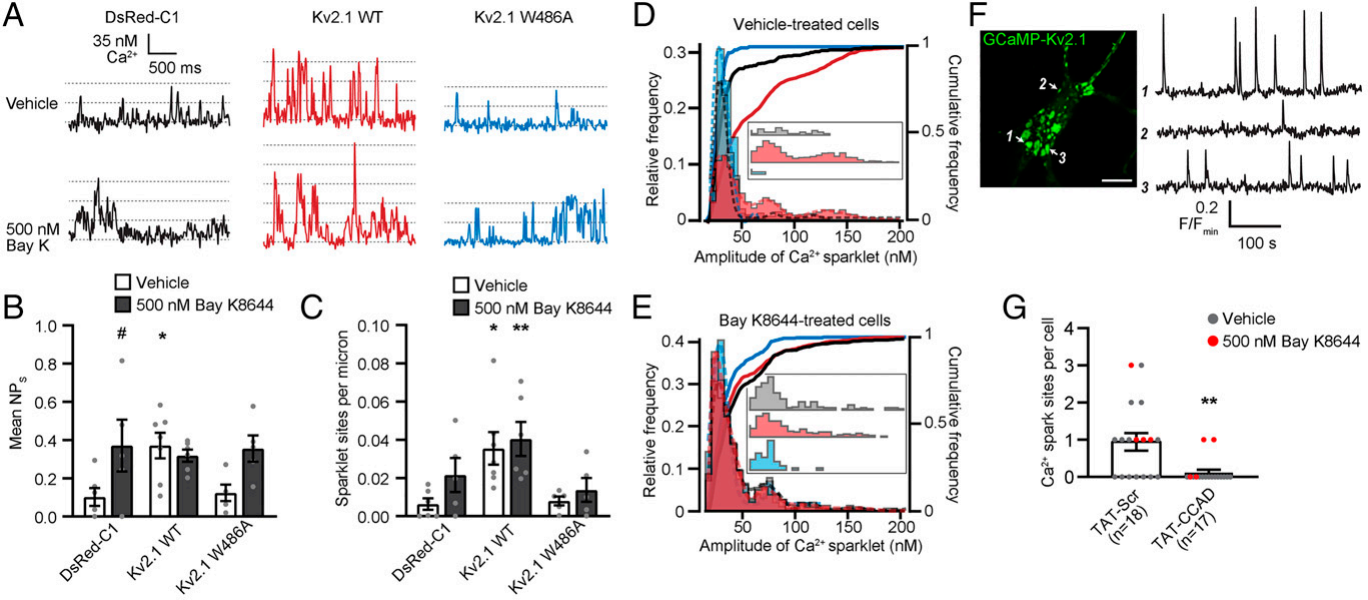
Disruption of the CCAD abolishes Kv2.1-dependent increases in endogenous LTCC activity. (*A*) [Ca^2+^]_i_ records from representative Ca^2+^ sparklet sites recorded in INS-1 cells transfected with the indicated constructs before (upper traces) and after (lower traces) treatment with 500 nM Bay K8644. (*B* and *C*) Bar graphs of mean ± SEM. NP_s_ (*B*) and sparklet density (*C*) before and after treatment with Bay K8644; (*B*: *P = 0.0297 and ^#^*P =* 0.051; *C*: *P = 0.0201 and **P = 0.0077; one-way ANOVA followed by Dunnett’s post hoc test versus DsRed-C1 vehicle). Each point represents a single cell. (*D* and *E*) All-points histograms and cumulative frequency distributions (solid lines) of Ca^2+^ sparklet data recorded from control cells (*D*) or cells treated with 500 nM Bay K8644 (*E*). *Insets* show unobscured histograms for multiquantal events (black: DsRed-C1; red: Kv2.1 WT; and blue: Kv2.1 W486A). The data were fit with a multicomponent Gaussian function (dashed lines). (*F*) Confocal image of a hippocampal neuron transfected with GCaMP3-Kv2.1. Arrows indicate Kv2.1 clusters displaying spontaneous Ca^2+^ sparks whose fluorescence intensity profiles are plotted in adjacent panel (Scale bar, 10 μm). (*G*) Quantification of Ca^2+^ sparks sites observed in neurons treated with TAT-CCAD or TAT-Scr peptides. Each point represents a single cell; bars are the average of mean experimental values ± SEM (**P = 0.0027; Student’s *t* test).

We next tested how these CCAD mutations and the TAT-CCAD cell-penetrating peptide impacted the function and localization of endogenous Cav1.2. We first employed an optical approach to measure single-Cav1.2 channel activity in the form of Ca^2+^ sparklets, which are local elevations in intracellular Ca^2+^ produced by the opening of a single or small cluster of Cav1.2 channels (49). We measured Ca^2+^ sparklets in INS-1 cells, a rat cell line derived from pancreatic β-cells with well-characterized Cav1.2-mediated Ca^2+^ currents (50). The compact morphology of these cells permitted effective control of the membrane potential to measure spontaneous openings of endogenous Cav1.2 channels at polarized membrane potentials. Similar to our previous observations with Cav1.2 channels expressed in HEK293T cells (35), we found that the expression of Kv2.1 WT (in the non–K^+^-conducting P404W background) in INS-1 cells increased sparklet activity (NP_s_) and the number of sparklet sites present in the TIRF footprint relative to control cells (Fig. 4 *A*–*C*). The fitting of multipeak Gaussians to the all-points histograms of the calibrated Ca^2+^ sparklet records (Fig. 4 *D* and *E*) indicated a quantal amplitude of 35.4 ± 2.3 nM Ca^2+^, similar to LTCC-mediated Ca^2+^ sparklets measured under similar conditions in other cell types (48, 51, 52). Cumulative frequency distribution plots revealed a greater frequency of multiquantal Ca^2+^ sparklets, corresponding to multiple simultaneous LTCC openings, in the DsRed-Kv2.1 group compared to the control DsRed or DsRed-Kv2.1 W486A groups (Fig. 4*D*). Moreover, the treatment of cells with the LTCC agonist Bay K8644 increased the Ca^2+^ sparklet activity of control DsRed and DsRed-Kv2.1 W486A groups but did not further enhance the activity of the DsRed-Kv2.1 group (Fig. 4 *B*, *C*, and *E*). These observations suggest that exogenously expressing Kv2.1 WT brought the endogenous Cav1.2 channels close to their maximum open probability, similar to results obtained for exogenous Cav1.2 channels coexpressed with Kv2.1 in HEK293T cells (35). In contrast, Ca^2+^ sparklets in cells expressing Kv2.1 W486A resembled those in cells lacking exogenous Kv2.1 expression, supporting the hypothesis that an intact CCAD is required for the Kv2.1-mediated potentiation of endogenous Cav1.2 channels.

We next examined how the TAT peptides impacted somatic LTCC-dependent Ca^2+^ signals in cultured rat hippocampal neurons transfected with GCaMP-Kv2.1. We previously determined that Ca^2+^ sparks reported by GCaMP-Kv2.1 are generated by the spontaneous opening of LTCCs juxtaposed to ER-localized RyRs at Kv2.1-associated ER-PM junctions (35). We found that treatment with TAT-CCAD occluded spontaneous Ca^2+^ sparks and reduced somatic Ca^2+^ sparks triggered by the treatment of cells with the LTCC activator Bay K8644 (Fig. 4 *F* and *G*). In contrast, more than half of cells treated with the control TAT-Scr peptide displayed spontaneous Ca^2+^ sparks. Together, these data suggest that the CCAD-mediated association of Cav1.2 with Kv2.1 enables the generation of LTCC- and RyR-mediated Ca^2+^ signals at somatic ER-PM junctions, and that the TAT-CCAD peptide interferes with the clustering of Cav1.2 at these sites.

**Fig. 5.**
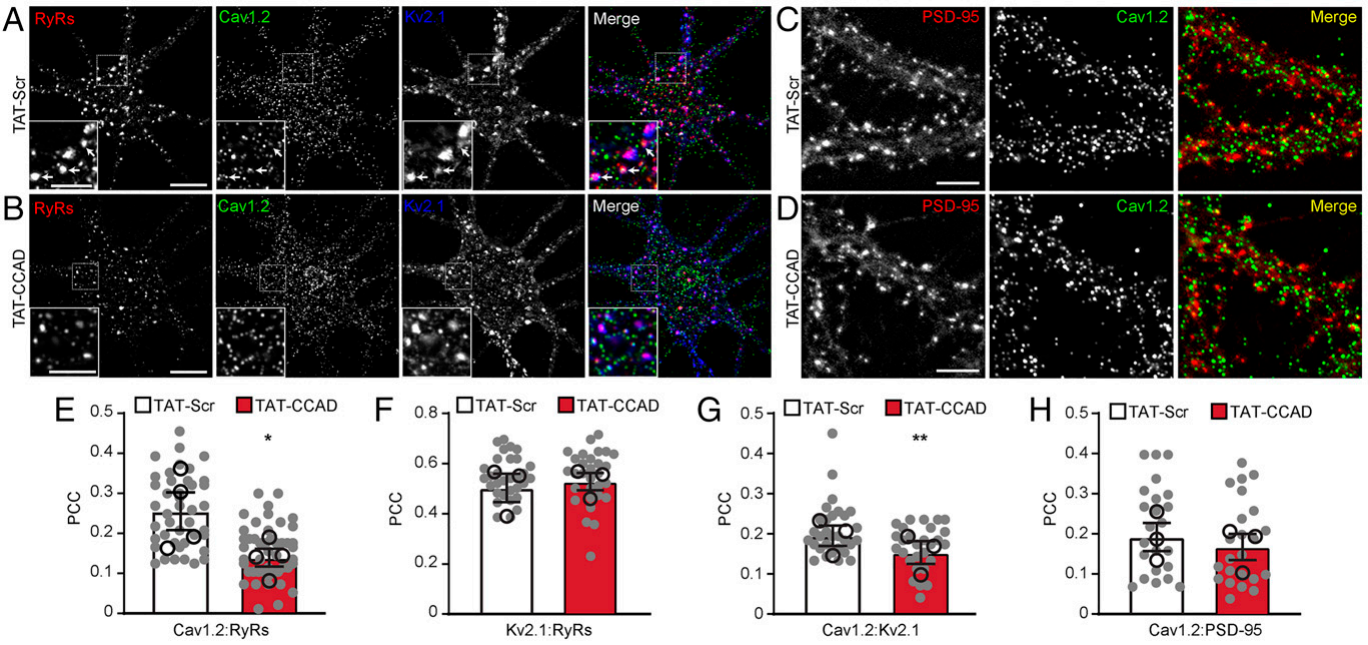
The Kv2.1 CCAD promotes the spatial coupling of LTCCs to ER-PM junctions in hippocampal neurons. (*A*) Representative images of the soma of a cultured hippocampal neuron treated overnight with 1 μM TAT-Scr and immunolabeled for RyRs (red), Cav1.2 (green), and Kv2.1 (blue). (Scale bar, 10 μm.) *Inset* shows an expanded view of the indicated region in the same neuron (Scale bar, 5 μm). Arrows in the *Inset* point to representative sites of RyRs, Cav1.2, and Kv2.1 in close proximity. (*B*) As in *A*, except in a neuron treated overnight with 1 μM TAT-CCAD. (*C*) Representative images of distal dendrites of cultured hippocampal neurons treated overnight with 1 μM TAT-Scr and immunolabeled for PSD-95 (red) and Cav1.2 (green). (Scale bar, 5 μm.) (*D*) As in *C*, except in neurons treated overnight with 1 μM TAT-CCAD. (*E*–*H*) PCC values of the immunofluorescence values between the indicated protein pairs in neurons treated with TAT-Scr or TAT-CCAD. Each point represents the PCC value from a single cell (solid circles) or the mean value from independent experiments (open circles); bars are the average of mean experimental values ± SEM (**P = 0.0055 and *P = 0.0498; paired *t* test).

We tested this possibility by assessing how the treatment of neurons with TAT peptides impacted the spatial coupling of somatic Cav1.2 channels with RyRs (35). We found that the TAT-CCAD treatment of hippocampal neurons decreased the colocalization of Cav1.2 with RyRs (Fig. 5 *A*, *B*, and *E*). In contrast, TAT-CCAD treatment did not impact the spatial coupling of Kv2.1 with RyRs (Fig. 5 *A*, *B*, *E*, and *F*). While treatment with the TAT-CCAD peptide reduced the association of Kv2.1 with Cav1.2 in the soma, it did not alter the localization of Cav1.2 in distal dendrites, as evidenced by the lack of an effect on the extent of colocalization of Cav1.2 with the postsynaptic protein PSD-95 (Fig. 5*H*). These results suggest that interfering with the function of the Kv2.1 CCAD selectively disrupts the Kv2.1-mediated localization of Cav1.2 channels at somatic ER-PM junctions. The findings that the TAT-CCAD peptide suppressed the Kv2.1-mediated increases in somatic Cav1.2 clustering and function, but had no impact on Cav1.2 alone or on its localization on distal dendrites that lack Kv2.1, suggest that it can be used as a selective modulator of somatic but not distal dendritic Cav1.2 channel function.

**Fig. 6.**
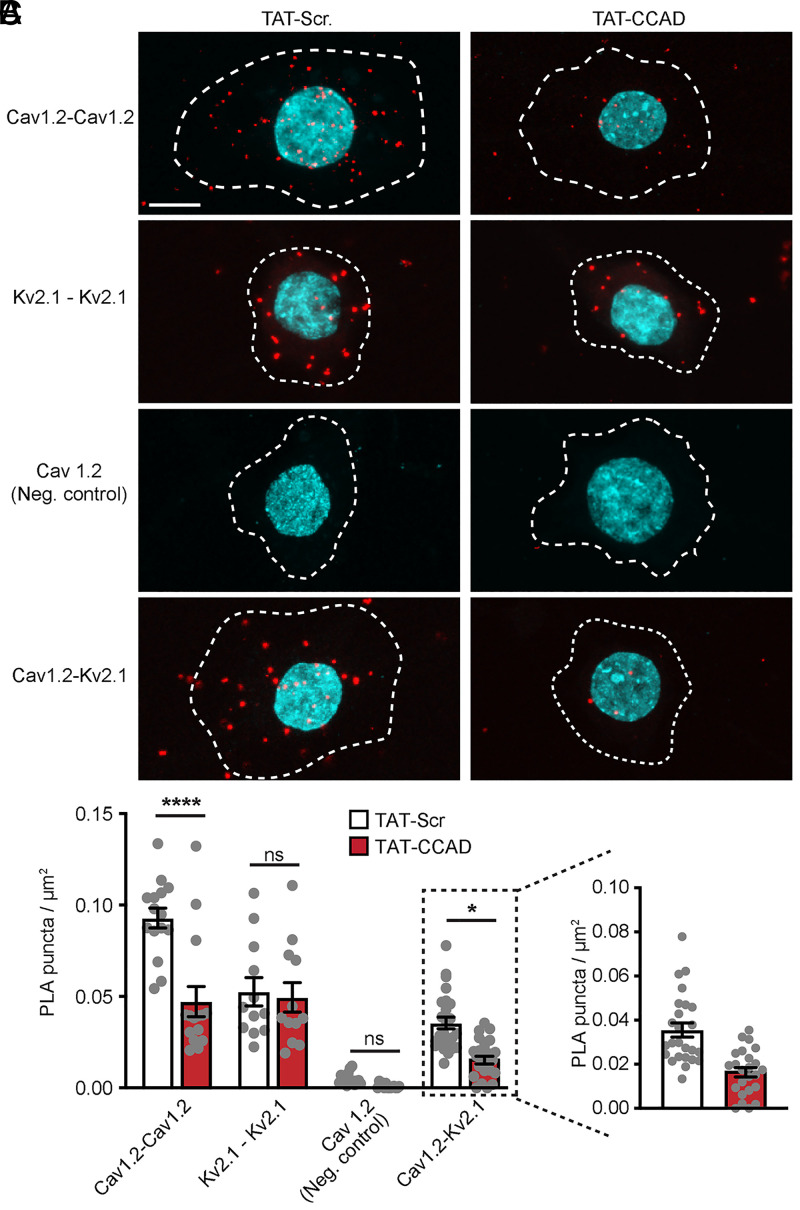
A cell-permeant fragment of the CCAD selectively disrupts the spatial association of Kv2.1 and Cav1.2 in hippocampal neurons. (*A*) Representative images of PLA puncta (red) generated between the indicated antibody pairs in cultured hippocampal neurons treated overnight with 1 μM TAT-Scr (*Left* column) or TAT-CCAD (*Right* column). Nuclei as labeled by Hoechst staining are shown in cyan. Dashed line demarcates the soma. (Scale bar, 10 μm.) (*B*) Quantification of PLA puncta per square micron of the somatic membrane; each point represents the mean number of puncta for a single cell; bars are mean ± SEM (****P < 0.0001, *P = 0.0152, and ns: not significant; one-way ANOVA followed by Tukey’s post hoc test). An expanded view of Kv2.1-Cav1.2 PLA puncta quantification is shown in *C*.

We next determined how treatment with the TAT-CCAD peptide influenced the spatial proximity of endogenous Kv2.1 and Cav1.2 channels to one another using a proximity ligation assay [PLA (53)] to detect Kv2.1 and Cav1.2 channels that are within 40 nm of each other (37). The overnight treatment of neurons with TAT-CCAD reduced the Kv2.1-Cav1.2 PLA signal, an effect not seen with TAT-Scr (Fig. 6 *A*–*C*), supporting that this Kv2.1-derived cell-penetrating peptide interferes with the association of endogenous Kv2.1 and Cav1.2 channels. Consistent with the model that the Kv2.1-mediated clustering of Cav1.2 increases the association of Cav1.2 channels with one another (37) and our split-Venus results in HEK293T cells (Fig. 3), TAT-CCAD treatment also reduced the Cav1.2-Cav1.2 PLA signal (Fig. 6 *A* and *B*). The Kv2.1-Kv2.1 PLA signal was not impacted by TAT-CCAD treatment (Fig. 6 *A* and *B*), consistent with imaging results in HEK293T cells (Figs. 1 and 2), showing that while the CCAD is necessary and sufficient for the association of Kv2.1 with Cav1.2, it is not involved in the clustering of Kv2.1 itself.

### Disruption of the Kv2.1 CCAD Suppresses Excitation–Transcription Coupling.

**Fig. 7.**
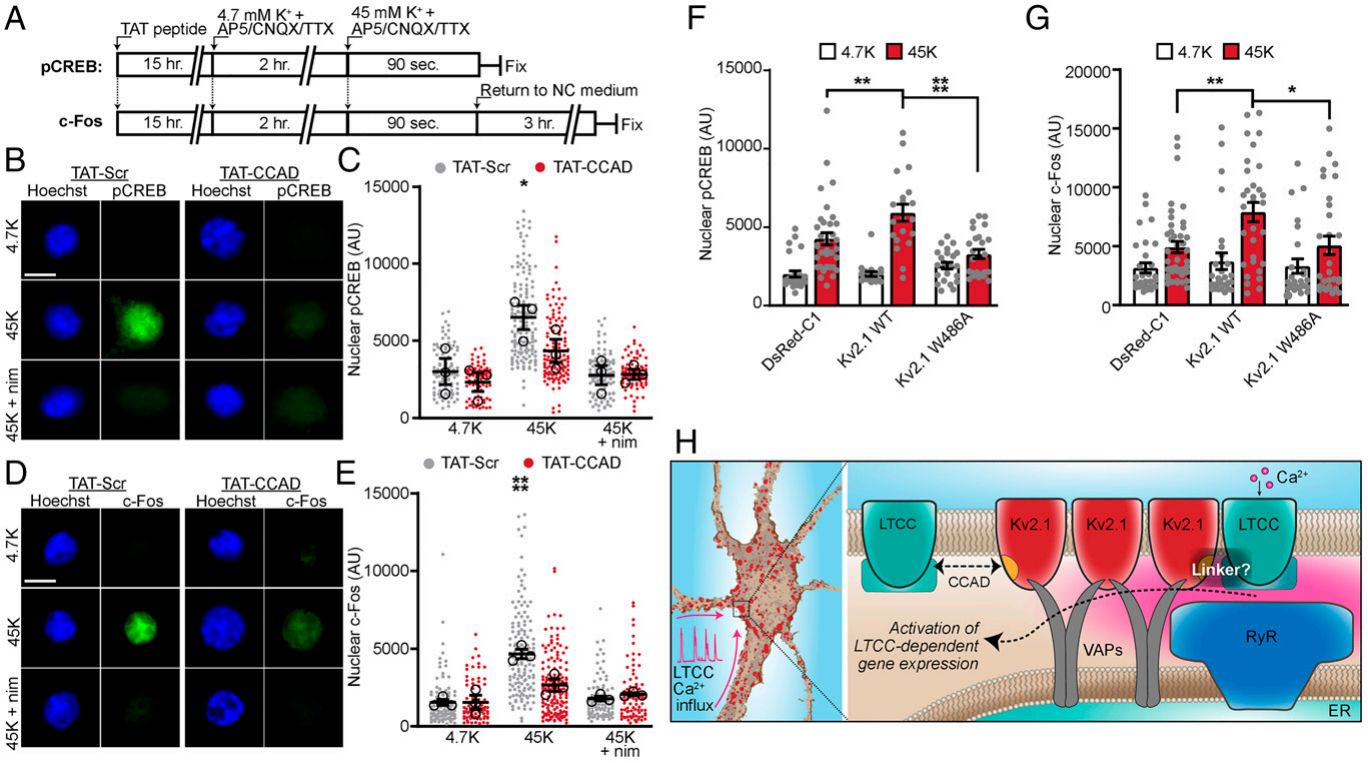
LTCCs associated with Kv2.1-mediated ER-PM junctions play a prominent role in excitation–transcription coupling. (*A*) Schematic of experimental protocols for 45 mM K^+^ depolarization-induced phosphorylation of CREB at S133 (pCREB) or the expression of c-Fos (NC medium: neuronal culture medium). (*B*) Representative images of nuclei and pCREB immunofluorescence in neurons treated with TAT-Scr or TAT-CCAD under each condition (4.7K: 4.7 mM K^+^; 45K: 45 mM K^+^; and 45K + nim: 45 mM K^+^ + 10 μM nimodipine) (Scale bar, 10 μm). (*C*) Nuclear pCREB levels in hippocampal neurons treated as indicated. Each point represents a single cell (solid circles) or the mean value from each of three independent experiments (open circles); bars are the average of mean experimental values ± SEM (*P = 0.0133; one-way ANOVA followed by Dunnett’s post hoc test versus TAT-Scr 4.7K). (*D*) As in *B*, but for c-Fos immunolabeling. (*E*) As in *C*, but for nuclear c-Fos (****P < 0.0001; one-way ANOVA followed by Dunnett’s post hoc test versus TAT-Scr 4.7K). (*F*) Nuclear pCREB levels in hippocampal neurons transfected with the indicated constructs and treated with 4.7 mM K^+^ or 45 mM K^+^. Each point represents a single cell; bars are mean ± SEM (****P < 0.0001 and **P = 0.0070; one-way ANOVA followed by Tukey’s post hoc test). (*G*) As in *F*, but for nuclear c-Fos levels (**P = 0.0079 and *P = 0.0295; one-way ANOVA followed by Tukey’s post hoc test). (*H*) Working model. The Kv2.1 CCAD promotes the targeting of LTCCs to somatic ER-PM junctions to enhance Ca^2+^ signals that trigger gene expression.

Neuronal LTCCs including Cav1.2 uniquely contribute to membrane depolarization-dependent changes in transcription factor activation by mediating Ca^2+^ influx within specialized and compartmentalized signaling complexes (3, 17, 54–57). Excitation-transcription coupling can be initiated at distal synaptic sites (58–60), although somatic depolarization in itself is sufficient (61) and certain forms require activation of somatic LTCCs (56). While much is known of the protein complexes involved in mediating Cav1.2-dependent forms of synaptic plasticity and excitation-transcription coupling triggered at distal synaptic sites (12, 62), the nature of signaling complexes underlying somatic LTCC-mediated excitation-transcription coupling has not been defined. We previously established that Kv2.1 organizes Cav1.2 in close proximity to somatic ER-PM junctions in hippocampal neurons (35). The results presented here indicate that disruption of the Kv2.1 CCAD permits selective decoupling of Cav1.2 from these sites. Therefore, we tested how selective disruption of Cav1.2 recruitment to Kv2-organized ER-PM junctions would impact depolarization-induced Cav1.2 Ca^2+^ influx-dependent changes in transcription factor activation and gene expression. Phosphorylation of the transcription factor CREB at S133 is triggered by Ca^2+^ influx through LTCCs and stimulates expression of immediate early genes encoding multiple proteins, including the transcription factor c-Fos, that serve important roles in learning and memory (17, 55, 63–65). We treated cultured hippocampal neurons with TAT peptides overnight and investigated how membrane potential depolarization induced by elevated extracellular K^+^ affected nuclear CREB phosphorylation and induction of c-Fos expression. We first silenced the synaptic and intrinsic activity of neurons for two hours using a buffer solution containing CNQX and AP5 to block glutamate receptors, and tetrodotoxin (TTX) to block voltage-gated Na^+^ channels (17); TAT-CCAD or TAT-Scr peptides were also included in this preincubation medium. Neurons were then depolarized for 90 seconds with 45 mM K^+^ buffer, also supplemented with CNQX, AP5, and TTX, predicted to depolarize the membrane potential to >−19 mV (17), to induce cell autonomous LTCC-dependent transcription factor activation (66) (Fig. 7*A*). We found that TAT-CCAD peptide treatment reduced the depolarization-triggered increases in the levels of nuclear pCREB (Fig. 7 *B* and *C*) and c-Fos expression (Fig. 7 *D* and *E*), supporting a critical role for LTCCs clustered at Kv2-associated ER-PM junctions in mediating LTCC excitation-transcription coupling. Treatment with TAT-Scr peptide was without effect. The impact of TAT-CCAD treatment was mimicked by treatment with the LTCC blocker nimodipine, which as previously reported also blocked induction of pCREB phosphorylation and c-Fos expression (66) (Fig. 7 *C* and *E*). Importantly, nimodipine occluded any further effects of the TAT-CCAD treatment on both pCREB and c-Fos induction (Fig. 7 *C* and *E*), showing that the effects of this cell penetrating peptide were mediated through its impact on LTCCs.

Given their impact in increasing Cav1.2 clustering and activity, we next evaluated whether exogenous expression of nonconducting Kv2.1 channels in hippocampal neurons could impact LTCC-dependent changes in pCREB or c-Fos. We performed the same high K^+^-induced depolarization assay using neurons transfected with nonconducting (i.e., in the P404W point mutant background) Kv2.1 WT. We found that expression of Kv2.1 WT in cultured neurons increased K^+^-depolarization-induced CREB phosphorylation (Fig. 7*F*) and c-Fos (Fig. 7*G*) expression relative to neurons expressing DsRed. However, hippocampal neurons expressing the Kv2.1 CCAD mutant W486A had levels of nuclear pCREB (Fig. 7*F*) and c-Fos (Fig. 7*G*) comparable to neurons lacking exogenous Kv2.1 expression. Together, these results support that the somatic population of LTCCs associated with Kv2.1 plays a prominent role in excitation-transcription coupling, and that Kv2.1-mediated increases in LTCC clustering and activity, and/or recruitment to ER-PM junctions regulate LTCC-dependent excitation-transcription coupling (Fig. 7*H*).

## Discussion

In this study, we made three fundamental observations regarding the organization of somatic Kv2.1, Cav1.2, and ER-PM junctions and their role in regulating excitation–transcription coupling. First, we discovered that Kv2.1-mediated ER-PM junction formation and the recruitment of Cav1.2 channels are separable functions of Kv2.1 mediated by distinct domains (the PRC and CCAD, respectively) within its C terminus. Second, the targeted disruption of the Kv2.1 CCAD impaired Kv2.1-dependent increases in Cav1.2 clustering and activity, and the association of Cav1.2 with ER-PM junctions, but unlike mutations in the PRC domain, did not impact the clustering of Kv2.1 at these sites. Third, the selective uncoupling of somatic LTCCs from Kv2.1-mediated ER-PM junctions in hippocampal neurons using CCAD mutants or a cell-penetrating interfering peptide suppressed LTCC-dependent excitation–transcription coupling in the form of depolarization-induced nuclear CREB phosphorylation and c-Fos expression. These findings suggest that the subset of Cav1.2 channels localized in specific microdomains at somatic Kv2.1-mediated ER-PM junctions play a key role in generating the Ca^2+^ signals that trigger depolarization-induced gene expression. Based on these results, we propose a model in which the CCAD links Kv2.1 to somatic Cav1.2 channels, promoting their clustering at ER-PM junctions, modulating their activity, and impacting their role in excitation–transcription coupling (Fig. 7*H*).

Our results show that LTCC-dependent excitation–transcription coupling was impaired by the selective, CCAD-dependent uncoupling of somatic Cav1.2 channels from Kv2.1-mediated ER-PM junctions. It has long been recognized that LTCCs play a privileged role in the Ca^2+^ influx mediating important forms of excitation–transcription coupling in brain neurons (63, 67, 68). However, for the most part, these studies were not able to distinguish the roles of the distinct populations of LTCCs on the neuronal soma and on distal dendrites near dendritic spines. Given its proximity to the nucleus, Ca^2+^ influx via somatic LTCCs is expected to be especially important for triggering gene expression (56, 61, 69, 70). Recently, additional experimental support for the concept of “soma to nucleus signaling” (61) was provided by studies employing neurons grown in microfluidic chambers that allowed for the separate interrogation of the contribution of the somatic and distal dendritic populations of LTCCs (56). These studies revealed a specific role for somatic LTCCs in mediating the Ca^2+^ influx underlying the calcineurin-dependent activation of the NFAT transcription factor (56). As Kv2.1 expression is restricted to the soma and proximal dendrites, the impact of the Kv2.1-mediated regulation of LTCCs would presumably be restricted to these same neuronal compartments and would not impact LTCCs on distal dendrites at/near dendritic spines. Our findings show that treatments with either the TAT-CCAD cell-penetrating peptide or the LTCC blocker nimodipine inhibit excitation–transcription coupling to a similar extent and that the treatments occlude one another. Taken together with the other results presented here, this provides additional evidence that LTCCs on the soma and proximal dendrites, and specifically those localized in specific microdomains at Kv2.1-containing ER-PM junctions, play a prominent role in mediating the Ca^2+^ increases that trigger excitation–transcription coupling. The Kv2.1-mediated increase in the localization of Cav1.2 with ER-PM junctions results in their increased spatial and functional association with ER-localized RyRs and more robust Ca^2+^-induced Ca^2+^ release at these sites (35), which could further amplify somatic Ca^2+^ signals and impact nuclear transcription factor activation. Decoupling LTCCs from Kv2.1 and somatic ER-PM junctions by CCAD interference could impair the LTCC-dependent activation of critical signaling proteins, reducing the subsequent phosphorylation of CREB and gene expression, although our data do not rule out additional effects on other components of the signaling pathway. The future use of the TAT-CCAD cell-penetrating peptide as a selective modulator of LTCCs on the soma and proximal dendrites, but not of those in distal dendrites at/near dendritic spines, could provide further insights into the contribution of these distinct populations of LTCCs in neuronal function and plasticity.

A key finding in our study is that Kv2.1, through its CCAD, plays a nonconducting structural role to regulate LTCCs that does not require its K^+^ conductance. Our data suggest that Kv2.1-Cav1.2 interactions require an intact CCAD and that the TAT-CCAD peptide interferes with this association. One limitation of our current study is that it does not reveal the precise molecular mechanism of this interaction, including whether it is direct or indirect. The CCAD sequence identified here has also been shown to be necessary for Kv2.1 binding to the SNARE protein Stx-1A (44–46), raising the possibility that Stx-1A may serve as an intermediary between Kv2.1 and LTCCs. However, in brain neurons, Stx-1A is primarily found in presynaptic terminals, where it is an integral part of the vesicle fusion complex (71) with little detectable expression on the somata of hippocampal neurons that exhibit prominent coclustering of Kv2.1 and LTCCs. Moreover, while we have identified VAP proteins (29) and numerous LTCC subunits (35) in our proteomic analyses of components of native brain Kv2.1-containing ion channel complexes, we have not detected Stx-1A. However, Cav1.2 has been shown to interact with Stx-1A in neuroendocrine cells (72, 73) and when heterologously expressed in HEK293 cells (74). These observations raise the possibility that in certain cell types Stx-1A may influence the association of Kv2.1 with Cav1.2 by acting as a binding intermediary, a competitive inhibitor, or in another role. The TAT-CCAD peptide has been previously shown to exhibit neuroprotective effects in in vitro and in vivo ischemic stroke models (44). Our data show that this cell-penetrating peptide reduces Cav1.2 channel activity and the spatial and functional association of Cav1.2 with RyRs at neuronal ER-PM junctions, sites that are also rich in mitochondria (75). Given the prominent role of increased intracellular Ca^2+^ and mitochondrial Ca^2+^ overload in ischemia-induced neuronal cell death (76), it will be of interest to determine whether the impact of TAT-CCAD peptide on suppressing Cav1.2-mediated Ca^2+^ signaling plays any role in its neuroprotective effects.

In summary, we have identified a domain in Kv2.1 that underlies its ability to promote the clustering of LTCCs at ER-PM junctions. This allowed us to selectively manipulate the clustering, activity, and function of somatic LTCCs and define their role in excitation–transcription coupling. These results not only provide further insights into the physiological nonconducting functions of Kv2.1 channels but also suggest an important role of LTCC-dependent Ca^2+^ signals at somatic ER-PM junctions in initiating depolarization-triggered gene transcription in neurons.

## Materials and Methods

Experimental procedures are described in detail in *SI Appendix*.

### Animals.

All procedures using mice and rats were approved by the University of California, Davis Institutional Animal Care and Use Committee and performed in accordance with the NIH *Guide for the Care and Use of Laboratory Animals* (77). Animals were maintained under standard light–dark cycles and allowed to feed and drink ad libitum. Experiments using hippocampal neurons were performed using cultures obtained from pooling neurons from Sprague-Dawley rat fetuses of both sexes. Adult male mice were used in immunohistochemistry experiments.

### Cell Culture, Transfection, and TAT Peptide Treatments.

HEK293T cells (ATCC No. CRL-3216) were cultured and transfected using Lipofectamine 2000 using standard procedures (details in *SI Appendix*). Neuronal cultures were prepared and maintained using a standard protocol (78) (details in *SI Appendix*). TAT-Scr and TAT-CCAD peptides were synthesized by GenScript at over 95% purity (peptide sequences are provided in *SI Appendix*, Table S2) and diluted to 1 μM in culture medium. For the evaluation of 45 mM K^+^-induced CREB S133 phosphorylation or c-Fos expression, DIV20 neurons were treated overnight with TAT peptides (details in *SI Appendix*).

### Immunolabeling and Imaging of Fixed Brain Sections, Cultured Neurons, and Cell Lines.

Preparation and immunolabeling of mouse brain sections was as previously described (27) (details in *SI Appendix*). Cultured neurons and HEK293T cells were immunolabeled as described previously (35) (details in *SI Appendix*).

### Live Cell Ca^2+^ Imaging and Bimolecular Fluorescence Complementation.

HEK293T cells transfected with the respective Cav channel subunits were imaged 48 h after transfection and after overnight treatments with TAT peptides (details in *SI Appendix*). Electrophysiological recordings were acquired at room temperature using an Axopatch200B amplifier and a Digidata1440 digitizer using pClamp 10.2 software (Molecular Devices) (details in *SI Appendix*).

### Sparklets.

Ca^2+^ sparklets were recorded using a dual-TIRF imaging/patch clamp system using INS-1 cells 48 h after transfection with respective Kv2.1 plasmids, employing Fluo-5F (Invitrogen No. F14221) as a Ca^2+^ reporter (details in *SI Appendix*).

### PLA.

The Duolink In Situ PLA kit (Sigma) was used to detect Cav1.2 and Kv2.1 in DIV20 neurons treated overnight with 1 μM TAT-CCAD or TAT-Scr and fixed 15 h later (details in *SI Appendix*).

### Experimental Design and Statistical Analysis.

For all datasets presented in this study for which statistical analyses were performed, measurements were imported into GraphPad Prism for statistical analysis and presentation. Reported values are mean ± SEM. Exact *P* values are reported in each figure legend. Paired datasets were compared using a Student’s *t* test if the data passed a normality test; a nonparametric test was used otherwise. For all experiments, at least two independent cultures were used for experimentation; the number of samples analyzed is noted in each figure or figure legend.

## Data Availability

All study data are included in the article and/or *SI Appendix*.

## References

[1] H. Ma, S. Cohen, B. Li, R. W. Tsien, Exploring the dominant role of Cav1 channels in signalling to the nucleus. Biosci. Rep. 33, 97–101 (2012). 23088728 10.1042/BSR20120099 PMC3546354

[2] F. Hofmann, V. Flockerzi, S. Kahl, J. W. Wegener, L-type CaV1.2 calcium channels: From in vitro findings to in vivo function. Physiol. Rev. 94, 303–326 (2014). 24382889 10.1152/physrev.00016.2013

[3] E.-L. Yap, M. E. Greenberg, Activity-regulated transcription: Bridging the gap between neural activity and behavior. Neuron 100, 330–348 (2018). 30359600 10.1016/j.neuron.2018.10.013 PMC6223657

[4] W. A. Catterall, Voltage-gated calcium channels. Cold Spring Harb. Perspect. Biol. 3, a003947 (2011). 21746798 10.1101/cshperspect.a003947 PMC3140680

[5] G. W. Zamponi, J. Striessnig, A. Koschak, A. C. Dolphin, The physiology, pathology, and pharmacology of voltage-gated calcium channels and their future therapeutic potential. Pharmacol. Rev. 67, 821–870 (2015). 26362469 10.1124/pr.114.009654 PMC4630564

[6] S. Moosmang , Role of hippocampal Cav1.2 Ca^2+^ channels in NMDA receptor-independent synaptic plasticity and spatial memory. J. Neurosci. 25, 9883–9892 (2005). 16251435 10.1523/JNEUROSCI.1531-05.2005 PMC6725564

[7] M. J. Sinnegger-Brauns , Expression and 1,4-dihydropyridine-binding properties of brain L-type calcium channel isoforms. Mol. Pharmacol. 75, 407–414 (2009). 19029287 10.1124/mol.108.049981

[8] J. W. Hell , Identification and differential subcellular localization of the neuronal class C and class D L-type calcium channel alpha 1 subunits. J. Cell Biol. 123, 949–962 (1993). 8227151 10.1083/jcb.123.4.949 PMC2200142

[9] N. Dedic , Cross-disorder risk gene CACNA1C differentially modulates susceptibility to psychiatric disorders during development and adulthood. Mol. Psychiatry 23, 533–543 (2018). 28696432 10.1038/mp.2017.133 PMC5822460

[10] D. G. Ehlinger, K. G. Commons, Cav1.2 L-type calcium channels regulate stress coping behavior via serotonin neurons. Neuropharmacology 144, 282–290 (2019). 30176250 10.1016/j.neuropharm.2018.08.033 PMC7476513

[11] S. J. Temme, R. Z. Bell, G. L. Fisher, G. G. Murphy, Deletion of the mouse homolog of CACNA1C disrupts discrete forms of hippocampal-dependent memory and neurogenesis within the dentate gyrus. eNeuro 3, ENEURO.0118-16.2016 (2016). 10.1523/ENEURO.0118-16.2016 PMC5124786 27957527

[12] S. Dai, D. D. Hall, J. W. Hell, Supramolecular assemblies and localized regulation of voltage-gated ion channels. Physiol. Rev. 89, 411–452 (2009). 19342611 10.1152/physrev.00029.2007 PMC2733249

[13] S. M. Cohen, B. Li, R. W. Tsien, H. Ma, Evolutionary and functional perspectives on signaling from neuronal surface to nucleus. Biochem. Biophys. Res. Commun. 460, 88–99 (2015). 25998737 10.1016/j.bbrc.2015.02.146 PMC4701207

[14] H. Ma , γCaMKII shuttles Ca^2+^/CaM to the nucleus to trigger CREB phosphorylation and gene expression. Cell 159, 281–294 (2014). 25303525 10.1016/j.cell.2014.09.019 PMC4201038

[15] J. G. Murphy , AKAP-anchored PKA maintains neuronal L-type calcium channel activity and NFAT transcriptional signaling. Cell Rep. 7, 1577–1588 (2014). 24835999 10.1016/j.celrep.2014.04.027 PMC4136445

[16] S. F. Oliveria, M. L. Dell’Acqua, W. A. Sather, AKAP79/150 anchoring of calcineurin controls neuronal L-type Ca^2+^ channel activity and nuclear signaling. Neuron 55, 261–275 (2007). 17640527 10.1016/j.neuron.2007.06.032 PMC2698451

[17] D. G. Wheeler, C. F. Barrett, R. D. Groth, P. Safa, R. W. Tsien, CaMKII locally encodes L-type channel activity to signal to nuclear CREB in excitation-transcription coupling. J. Cell Biol. 183, 849–863 (2008). 19047462 10.1083/jcb.200805048 PMC2592819

[18] P. J. Dittmer, A. R. Wild, M. L. Dell’Acqua, W. A. Sather, STIM1 Ca^2+^ sensor control of L-type Ca^2+^-channel-dependent dendritic spine structural plasticity and nuclear signaling. Cell Rep. 19, 321–334 (2017). 28402855 10.1016/j.celrep.2017.03.056 PMC5451256

[19] B. Li, M. R. Tadross, R. W. Tsien, Sequential ionic and conformational signaling by calcium channels drives neuronal gene expression. Science 351, 863–867 (2016). 26912895 10.1126/science.aad3647 PMC5467645

[20] E. Nanou, W. A. Catterall, Calcium channels, synaptic plasticity, and neuropsychiatric disease. Neuron 98, 466–481 (2018). 29723500 10.1016/j.neuron.2018.03.017

[21] S. Wang , Densin-180 controls the trafficking and signaling of L-type voltage-gated Ca_v_1.2 Ca^2+^ channels at excitatory synapses. J. Neurosci. 37, 4679–4691 (2017). 28363979 10.1523/JNEUROSCI.2583-16.2017 PMC5426563

[22] N. Spruston, C. McBain, “Structural and functional properties of hippocampal neurons” in The Hippocampus Book, P. Andersen, R. Morris, D. Amaral, T. Bliss, J. O’Keefe, Eds. (Oxford University Press, New York, 2007), pp. 133–201.

[23] J. S. Trimmer, Immunological identification and characterization of a delayed rectifier K^+^ channel polypeptide in rat brain. Proc. Natl. Acad. Sci. U.S.A. 88, 10764–10768 (1991). 1961744 10.1073/pnas.88.23.10764 PMC53011

[24] J. Du, J. H. Tao-Cheng, P. Zerfas, C. J. McBain, The K^+^ channel, Kv2.1, is apposed to astrocytic processes and is associated with inhibitory postsynaptic membranes in hippocampal and cortical principal neurons and inhibitory interneurons. Neuroscience 84, 37–48 (1998). 9522360 10.1016/s0306-4522(97)00519-8

[25] T. Kirizs, K. Kerti-Szigeti, A. Lorincz, Z. Nusser, Distinct axo-somato-dendritic distributions of three potassium channels in CA1 hippocampal pyramidal cells. Eur. J. Neurosci. 39, 1771–1783 (2014). 24606584 10.1111/ejn.12526 PMC4150533

[26] D. Mandikian , Cell type-specific spatial and functional coupling between mammalian brain Kv2.1 K^+^ channels and ryanodine receptors. J. Comp. Neurol. 522, 3555–3574 (2014). 24962901 10.1002/cne.23641 PMC4139460

[27] H. I. Bishop , Distinct cell- and layer-specific expression patterns and independent regulation of Kv2 channel subtypes in cortical pyramidal neurons. J. Neurosci. 35, 14922–14942 (2015). 26538660 10.1523/JNEUROSCI.1897-15.2015 PMC4635138

[28] H. I. Bishop , Kv2 ion channels determine the expression and localization of the associated AMIGO-1 cell adhesion molecule in adult brain neurons. Front. Mol. Neurosci. 11, 1 (2018). 29403353 10.3389/fnmol.2018.00001 PMC5780429

[29] M. Kirmiz, N. C. Vierra, S. Palacio, J. S. Trimmer, Identification of VAPA and VAPB as Kv2 channel-interacting proteins defining endoplasmic reticulum-plasma membrane junctions in mammalian brain neurons. J. Neurosci. 38, 7562–7584 (2018). 30012696 10.1523/JNEUROSCI.0893-18.2018 PMC6113906

[30] B. Johnson , Kv2 potassium channels form endoplasmic reticulum/plasma membrane junctions via interaction with VAPA and VAPB. Proc. Natl. Acad. Sci. U.S.A. 115, E7331–E7340 (2018). 29941597 10.1073/pnas.1805757115 PMC6077746

[31] M. Kirmiz , Remodeling neuronal ER-PM junctions is a conserved nonconducting function of Kv2 plasma membrane ion channels. Mol. Biol. Cell 29, 2410–2432 (2018). 30091655 10.1091/mbc.E18-05-0337 PMC6233057

[32] C. G. F. de Kovel , Neurodevelopmental disorders caused by de novo variants in KCNB1 genotypes and phenotypes. JAMA Neurol. 74, 1228–1236 (2017). 28806457 10.1001/jamaneurol.2017.1714 PMC5710242

[33] C. Bar , Expanding the genetic and phenotypic relevance of KCNB1 variants in developmental and epileptic encephalopathies: 27 new patients and overview of the literature. Hum. Mutat. 41, 69–80 (2020). 31513310 10.1002/humu.23915

[34] C. J. Stefan, Endoplasmic reticulum-plasma membrane contacts: Principals of phosphoinositide and calcium signaling. Curr. Opin. Cell Biol. 63, 125–134 (2020). 32088611 10.1016/j.ceb.2020.01.010

[35] N. C. Vierra, M. Kirmiz, D. van der List, L. F. Santana, J. S. Trimmer, Kv2.1 mediates spatial and functional coupling of L-type calcium channels and ryanodine receptors in mammalian neurons. eLife 8, e49953 (2019). 31663850 10.7554/eLife.49953 PMC6839919

[36] D. E. Antonucci, S. T. Lim, S. Vassanelli, J. S. Trimmer, Dynamic localization and clustering of dendritic Kv2.1 voltage-dependent potassium channels in developing hippocampal neurons. Neuroscience 108, 69–81 (2001). 10.1016/s0306-4522(01)00476-6 11738132

[37] S. C. O’Dwyer , Kv2.1 channels play opposing roles in regulating membrane potential, Ca^2+^ channel function, and myogenic tone in arterial smooth muscle. Proc. Natl. Acad. Sci. U.S.A. 117, 3858–3866 (2020). 32015129 10.1073/pnas.1917879117 PMC7035623

[38] R. E. Dixon, C. Yuan, E. P. Cheng, M. F. Navedo, L. F. Santana, Ca^2+^ signaling amplification by oligomerization of L-type Cav1.2 channels. Proc. Natl. Acad. Sci. U.S.A. 109, 1749–1754 (2012). 22307641 10.1073/pnas.1116731109 PMC3277143

[39] R. H. Scannevin, H. Murakoshi, K. J. Rhodes, J. S. Trimmer, Identification of a cytoplasmic domain important in the polarized expression and clustering of the Kv2.1 K^+^ channel. J. Cell Biol. 135, 1619–1632 (1996). 8978827 10.1083/jcb.135.6.1619 PMC2133974

[40] S. T. Lim, D. E. Antonucci, R. H. Scannevin, J. S. Trimmer, A novel targeting signal for proximal clustering of the Kv2.1 K^+^ channel in hippocampal neurons. Neuron 25, 385–397 (2000). 10719893 10.1016/s0896-6273(00)80902-2

[41] A. B. Gussow, S. Petrovski, Q. Wang, A. S. Allen, D. B. Goldstein, The intolerance to functional genetic variation of protein domains predicts the localization of pathogenic mutations within genes. Genome Biol. 17, 9 (2016). 26781712 10.1186/s13059-016-0869-4 PMC4717634

[42] D. P. Mohapatra, D. F. Siino, J. S. Trimmer, Interdomain cytoplasmic interactions govern the intracellular trafficking, gating, and modulation of the Kv2.1 channel. J. Neurosci. 28, 4982–4994 (2008). 18463252 10.1523/JNEUROSCI.0186-08.2008 PMC3409667

[43] X. Q. Dai , The voltage-dependent potassium channel subunit Kv2.1 regulates insulin secretion from rodent and human islets independently of its electrical function. Diabetologia 55, 1709–1720 (2012). 22411134 10.1007/s00125-012-2512-6

[44] C.-Y. Yeh , Targeting a potassium channel/syntaxin interaction ameliorates cell death in ischemic stroke. J. Neurosci. 37, 5648–5658 (2017). 28483976 10.1523/JNEUROSCI.3811-16.2017 PMC5469303

[45] M. C. McCord , Syntaxin-binding domain of Kv2.1 is essential for the expression of apoptotic K+ currents. J. Physiol. 592, 3511–3521 (2014). 24928958 10.1113/jphysiol.2014.276964 PMC4229345

[46] C.-Y. Yeh , Defining the Kv2.1-syntaxin molecular interaction identifies a first-in-class small molecule neuroprotectant. Proc. Natl. Acad. Sci. U.S.A. 116, 15696–15705 (2019). 31308225 10.1073/pnas.1903401116 PMC6681760

[47] C. Bechara, S. Sagan, Cell-penetrating peptides: 20 years later, where do we stand? FEBS Lett. 587, 1693–1702 (2013). 23669356 10.1016/j.febslet.2013.04.031

[48] C. M. Moreno , Ca^2+^ entry into neurons is facilitated by cooperative gating of clustered Ca_V_1.3 channels. eLife 5, e15744 (2016). 27187148 10.7554/eLife.15744 PMC4869912

[49] H. Cheng, W. J. Lederer, Calcium sparks. Physiol. Rev. 88, 1491–1545 (2008). 18923188 10.1152/physrev.00030.2007

[50] M. D. Nitert, C. L. Nagorny, A. Wendt, L. Eliasson, H. Mulder, Ca_V_1.2 rather than Ca_V_1.3 is coupled to glucose-stimulated insulin secretion in INS-1 832/13 cells. J. Mol. Endocrinol. 41, 1–11 (2008). 18562674 10.1677/JME-07-0133

[51] M. F. Navedo, G. C. Amberg, M. Nieves, J. D. Molkentin, L. F. Santana, Mechanisms underlying heterogeneous Ca^2+^ sparklet activity in arterial smooth muscle. J. Gen. Physiol. 127, 611–622 (2006). 16702354 10.1085/jgp.200609519 PMC2151539

[52] R. E. Dixon , Graded Ca^2+^/calmodulin-dependent coupling of voltage-gated Ca_V_1.2 channels. eLife 4, e05608 (2015). 10.7554/eLife.05608 PMC4360655 25714924

[53] M. Gullberg , Cytokine detection by antibody-based proximity ligation. Proc. Natl. Acad. Sci. U.S.A. 101, 8420–8424 (2004). 15155907 10.1073/pnas.0400552101 PMC420409

[54] R. E. Dolmetsch, U. Pajvani, K. Fife, J. M. Spotts, M. E. Greenberg, Signaling to the nucleus by an L-type calcium channel-calmodulin complex through the MAP kinase pathway. Science 294, 333–339 (2001). 11598293 10.1126/science.1063395

[55] D. G. Wheeler , Ca(V)1 and Ca(V)2 channels engage distinct modes of Ca(^2+^) signaling to control CREB-dependent gene expression. Cell 149, 1112–1124 (2012). 22632974 10.1016/j.cell.2012.03.041 PMC3654514

[56] A. R. Wild , Synapse-to-nucleus communication through NFAT is mediated by L-type Ca^2+^ channel Ca^2+^ spike propagation to the soma. Cell Rep. 26, 3537–3550.e4 (2019). 30917310 10.1016/j.celrep.2019.03.005 PMC6521872

[57] J. G. Murphy, K. C. Crosby, P. J. Dittmer, W. A. Sather, M. L. Dell’Acqua, AKAP79/150 recruits the transcription factor NFAT to regulate signaling to the nucleus by neuronal L-type Ca^2+^ channels. Mol. Biol. Cell 30, 1743–1756 (2019). 31091162 10.1091/mbc.E19-01-0060 PMC6727748

[58] K. Deisseroth, H. Bito, R. W. Tsien, Signaling from synapse to nucleus: Postsynaptic CREB phosphorylation during multiple forms of hippocampal synaptic plasticity. Neuron 16, 89–101 (1996). 8562094 10.1016/s0896-6273(00)80026-4

[59] S. M. Dudek, R. D. Fields, Mitogen-activated protein kinase/extracellular signal-regulated kinase activation in somatodendritic compartments: Roles of action potentials, frequency, and mode of calcium entry. J. Neurosci. 21, RC122 (2001). 11160456 10.1523/JNEUROSCI.21-02-j0002.2001 PMC6763808

[60] G. E. Hardingham, F. J. Arnold, H. Bading, Nuclear calcium signaling controls CREB-mediated gene expression triggered by synaptic activity. Nat. Neurosci. 4, 261–267 (2001). 11224542 10.1038/85109

[61] S. M. Dudek, R. D. Fields, Somatic action potentials are sufficient for late-phase LTP-related cell signaling. Proc. Natl. Acad. Sci. U.S.A. 99, 3962–3967 (2002). 11891337 10.1073/pnas.062510599 PMC122631

[62] T. Patriarchi, O. R. Buonarati, J. W. Hell, Postsynaptic localization and regulation of AMPA receptors and Cav1.2 by β2 adrenergic receptor/PKA and Ca^2+^/CaMKII signaling. EMBO J. 37, e99771 (2018). 30249603 10.15252/embj.201899771 PMC6187224

[63] H. Bading, D. D. Ginty, M. E. Greenberg, Regulation of gene expression in hippocampal neurons by distinct calcium signaling pathways. Science 260, 181–186 (1993). 8097060 10.1126/science.8097060

[64] C. P. Bengtson, H. Bading, Nuclear calcium signaling. Adv. Exp. Med. Biol. 970, 377–405 (2012). 22351065 10.1007/978-3-7091-0932-8_17

[65] A. Ghosh, D. D. Ginty, H. Bading, M. E. Greenberg, Calcium regulation of gene expression in neuronal cells. J. Neurobiol. 25, 294–303 (1994). 7910846 10.1002/neu.480250309

[66] T. L. Perfitt , Neuronal L-type calcium channel signaling to the nucleus requires a novel CaMKIIα-Shank3 interaction. J. Neurosci. 40, 2000–2014 (2020). 32019829 10.1523/JNEUROSCI.0893-19.2020 PMC7055140

[67] T. H. Murphy, P. F. Worley, J. M. Baraban, L-type voltage-sensitive calcium channels mediate synaptic activation of immediate early genes. Neuron 7, 625–635 (1991). 1657056 10.1016/0896-6273(91)90375-a

[68] G. S. Brigidi , Genomic decoding of neuronal depolarization by stimulus-specific NPAS4 heterodimers. Cell 179, 373–391.e27 (2019). 31585079 10.1016/j.cell.2019.09.004 PMC6800120

[69] P. L. Greer, M. E. Greenberg, From synapse to nucleus: Calcium-dependent gene transcription in the control of synapse development and function. Neuron 59, 846–860 (2008). 18817726 10.1016/j.neuron.2008.09.002

[70] M. Matamales, Neuronal activity-regulated gene transcription: How are distant synaptic signals conveyed to the nucleus? F1000 Res. 1, 69 (2012). 10.12688/f1000research.1-69.v1 PMC3752646 24327840

[71] Y. Hata, C. A. Slaughter, T. C. Südhof, Synaptic vesicle fusion complex contains unc-18 homologue bound to syntaxin. Nature 366, 347–351 (1993). 8247129 10.1038/366347a0

[72] Y. M. Leung , Syntaxin 1A binds to the cytoplasmic C terminus of Kv2.1 to regulate channel gating and trafficking. J. Biol. Chem. 278, 17532–17538 (2003). 12621036 10.1074/jbc.M213088200

[73] L. Feinshreiber , Non-conducting function of the Kv2.1 channel enables it to recruit vesicles for release in neuroendocrine and nerve cells. J. Cell Sci. 123, 1940–1947 (2010). 20484665 10.1242/jcs.063719

[74] J. Sajman, M. Trus, D. Atlas, E. Sherman, The L-type voltage-gated calcium channel co-localizes with syntaxin 1A in nano-clusters at the plasma membrane. Sci. Rep. 7, 11350 (2017). 28900128 10.1038/s41598-017-10588-4 PMC5595989

[75] J. Rosenbluth, Subsurface cisterns and their relationship to the neuronal plasma membrane. J. Cell Biol. 13, 405–421 (1962). 14493991 10.1083/jcb.13.3.405 PMC2106078

[76] D. Bano, M. Ankarcrona, Beyond the critical point: An overview of excitotoxicity, calcium overload and the downstream consequences. Neurosci. Lett. 663, 79–85 (2018). 28843346 10.1016/j.neulet.2017.08.048

[77] National Research Council, Guide for the Care and Use of Laboratory Animals (National Academies Press, Washington, DC, ed. 8, 2011).

[78] S. Kaech, G. Banker, Culturing hippocampal neurons. Nat. Protoc. 1, 2406–2415 (2006). 17406484 10.1038/nprot.2006.356

